# CNS imaging characteristics in fibromyalgia patients with and without peripheral nerve involvement

**DOI:** 10.1038/s41598-022-10489-1

**Published:** 2022-04-25

**Authors:** Hans-Christoph Aster, Dimitar Evdokimov, Alexandra Braun, Nurcan Üçeyler, Thomas Kampf, Mirko Pham, György A. Homola, Claudia Sommer

**Affiliations:** 1 Neurologische Klinik und Poliklinik, Universitätsklinikum, Josef-Schneider-Str. 11, 97080 Würzburg, Germany; 2 Klinik für Kinder- und Jugendpsychiatrie, Psychotherapie und Psychosomatik, Margarate-Höppel-Platz 1, 97080 Würzburg, Germany; 3 Institut für Diagnostische und Interventionelle Neuroradiologie, Universitätsklinikum, Würzburg, Germany

**Keywords:** Neuropathic pain, Fibromyalgia

## Abstract

We tested the hypothesis that reduced skin innervation in fibromyalgia syndrome is associated with specific CNS changes. This prospective case–control study included 43 women diagnosed with fibromyalgia syndrome and 40 healthy controls. We further compared the fibromyalgia subgroups with reduced (n = 21) and normal (n = 22) skin innervation. Brains were analysed for cortical volume, for white matter integrity, and for functional connectivity. Compared to controls, cortical thickness was decreased in regions of the frontal, temporal and parietal cortex in the fibromyalgia group as a whole, and decreased in the bilateral pericalcarine cortices in the fibromyalgia subgroup with reduced skin innervation. Diffusion tensor imaging revealed a significant increase in fractional anisotropy in the corona radiata, the corpus callosum, cingulum and fornix in patients with fibromyalgia compared to healthy controls and decreased FA in parts of the internal capsule and thalamic radiation in the subgroup with reduced skin innervation. Using resting-state fMRI, the fibromyalgia group as a whole showed functional hypoconnectivity between the right midfrontal gyrus and the posterior cerebellum and the right crus cerebellum, respectively. The subgroup with reduced skin innervation showed hyperconnectivity between the inferior frontal gyrus, the angular gyrus and the posterior parietal gyrus. Our results suggest that the subgroup of fibromyalgia patients with pronounced pathology in the peripheral nervous system shows alterations in morphology, structural and functional connectivity also at the level of the encephalon. We propose considering these subgroups when conducting clinical trials.

## Introduction

The fibromyalgia syndrome (FMS) is a chronic pain disorder with a prevalence of approximately 2% in the general population^1^. Abnormalities in pain processing regions in the CNS, neurotransmitter levels, the autonomic nervous system, and in small fibers of the peripheral nervous system are frequent findings associated with FMS, but their causal connection to the manifestation and course of its symptoms is still unclear. Altered pain processing at the level of the CNS is regarded as a major pathophysiological factor^2,3^. However, structural lesions and functional deficits were also observed at the level of the PNS, where specifically small fiber pathology is a robust finding in a substantial group of patients fulfilling the established diagnostic criteria of FMS^4^. These findings of structural and functional alterations in FMS at both CNS and PNS level were reproducible: CNS structural measurements, like voxel-based-morphometry or cortical reconstruction, have revealed atrophy of the grey matter in the left prefrontal cortex and the posterior cingulate cortex^5,6^. Diffusion tensor imaging (DTI) has shown changes in white matter integrity, e.g. in the corpus callosum^7^, and functional magnetic resonance imaging (fMRI) has identified hyperactivity in many regions related to pain processing^8^, such as the left prefrontal cortex and in the posterior cingulate cortex, the insular cortex and the cerebellum. Functional connectivity was increased in the default mode network (DMN) and pain related areas, such as the insular cortex^9–11^. In the PNS, we and other groups described a decrease in intraepidermal nerve fiber density (IENFD)^12–17^, which was related to symptom severity^4^.

The relative importance of CNS and PNS abnormalities for FMS pathophysiology has been a matter of debate. A continuum between peripherally driven pain at one end and centrally driven pain at the other end has been suggested^3^. Whether CNS and PNS abnormalities coexist in the same patients, or whether CNS and PNS pathology define two non-overlapping subgroups in FMS has never been studied and presents a particular methodological challenge. For FMS, we addressed this challenge in the following manner: We established robust differences between two cohorts of FMS patients using objective and validated criteria of injury at the PNS level (FMS with markedly reduced IENFD vs. FMS with normal IENFD). We hypothesized that structural or functional remodeling of the brain would occur differentially in these two subgroups on a global or regional level. We tested this hypothesis in these two FMS subgroups versus case matched healthy controls using MRI methods to measure brain morphometry, structural and functional connectivity.

## Materials and methods

### Subjects

Forty-three female patients with FMS were recruited at the Department of Neurology, University Hospital Würzburg, who also had taken part in a previous study investigating small fiber pathology in FMS^4^. Forty healthy female age and sex matched controls were recruited via public announcements. All patients had been diagnosed with FMS and examined by a rheumatologist and a neurologist, fulfilled the diagnostic criteria for FMS according to the guidelines released by the American College of Rheumatology^18^, and had been comprehensively examined in our hospital for possible differential diagnoses (see Evdokimov et al. 2019^4^). Specifically, patients must have had widespread pain for more than three months that could not be explained by other diseases, have a Widespread Pain Index (WPI) ≥ 7 and the Symptom Severity Score ≥ 5^18^.

All patients were off their pain medication for 3 days before the examination. None of the patients and controls had been taking anticonvulsants, antihistamines, muscle relaxants or benzodiazepines within the 4 weeks before the examination. All participants in the study gave there written informed consent according to Declaration of Helsinki. The study was approved by the Ethics Committee of the University of Würzburg Medical Faculty (63/18). The exclusion criteria for patients and controls were other current autoimmune or inflammatory diseases that can cause pain, such as rheumatoid arthritis, systemic lupus erythematosus, or chronic inflammatory bowel disease, as well as neurological, cardiovascular, psychiatric diseases, such as major depression, in the past and at present, any contraindication for MRI like cardiac pacemakers, cochlear implants, vascular stents or metal splinters in the body, a history of drug abuse, a history of head trauma requiring medical attention or brains with significant structural abnormalities.

### Subgrouping according to intraepidermal nerve fiber density

Patients from the previous study^4^ who had either normal IENFD at the lower leg (above the lower limit of normal 5.4 fibers/mm) and at the upper thigh (above the lower limit of normal 8.5 fibers/mm) or a non-length dependent abnormal IENFD, which means the IENFD was below the lower limits at both biopsy sites, were re-recruited, i.e. were contacted by H.-C. A. and invited to a follow-up appointment for MRI imaging. The first group was termed “noPNS”, the second group “PNS”. These cut-off values were determined based on skin biopsies of these two regions of 120 healthy women (median age = 50 years, range = 20–84 years) in our department. The cut-off values represent the lower limit of the standard deviation of the IENFD results of all the healthy controls investigated in our laboratory.

### Fibromyalgia related symptoms

Results of the questionnaire and clinical examination data of the FMS patients have already been published^4^. To evaluate pain severity, two pain scores were used (Graded Chronic Pain Scale (GCPS) and Neuropathic Pain Symptom Inventory (NPSI)). In order to assess the depressiveness of the patients, the “Allgemeine Depressionskala” (ADS) was used, which is a German version of the Center for Epidemiological Studies—Depression scale questionnaire^19^. To evaluate catastrophizing, the Pain Catastrophizing Scale (PCS)^20^, which is a self-report measure, consisting of 13 items scored from 0 to 4, resulting in a total possible score of 52, was assessed. To test the anxiety level, the State-Trait Anxiety Inventory (STAI) was used^21^, which is a commonly used measure of trait and state anxiety. In order to assess the influence of the disease on daily experience, the Fibromyalgia Impact Questionnaire (FIQ)^22^ was used. Also, the Symptom Severity Scale (SSS) was used to query other FMS-associated symptoms^18^. It measures three key symptoms during the past week: Fatigue, unrefreshed wakening and cognitive impairment. The O’Leary-Sant Symptom and Problem Index assesses the impairment by bladder dysfunction^23^ and was selected, as FMS patients frequently report abdominal pain and problems with urination. Data collected in the context of the clinical diagnostics, such as the conduction studies of the sural nerve and the blood values, for example HbA1c and vitamin D, were also analyzed.

### MR imaging and analysis

#### Data acquisition

Magnetic resonance imaging was performed on a Siemens MAGNETOM Prisma fit Scanner (Siemens Healthcare GmbH, Erlangen, Germany), operating at 3 T, equipped with a 64-channel head coil at the Department of Neuroradiology, University Hospital Würzburg. For each participant we included a structural T1-weighted (T1w) sequence, diffusion weighted imaging (DWI), fieldmap data and resting-state functional MRI (rs-fMRI) series. The T1w gradient echo MPRAGE sequence (repetition time (TR) 2400 ms, echo time (TE) 3.17 ms, flip angle (FA) 8°, inversion recovery (IR) 1000 ms) contained 176 sagittal slices with an isotropic voxel size of 1 × 1 × 1 mm. The visual examination of the T1w-structural images revealed no gross morphological abnormalities for any patient or subject. DWI was obtained using multiband echo-planar imaging (EPI) with the following parameters: TR = 3100 ms, TE = 89 ms, FA = 90°, isotropic voxel size of 2 × 2 × 2 mm. Diffusion data were collected with reversed phase-encode blips, resulting in pairs of b0-images with distortions in opposite directions for further susceptibility induced distortion correction. Resting state fMRI data was acquired using a T2*-weighted multiband EPI sequence with TR = 1610 ms, TE = 30 ms, FA = 70°, isotropic voxel size of 2 × 2 × 2 mm, 69 slices. During the 9-min resting state fMRI acquisition period with 300 volumes the subjects were told to lie still and remain awake with their eyes open. Participants’ motion was minimized using tight foam pads around the head, their physiology was monitored.

#### Structural analysis

Cortical reconstruction and volumetric segmentation was performed with the FreeSurfer image analysis suite v6.0.0 (Martinos Center for Biomedical Imaging, Boston, MA, USA) using the 3D T1w data. The technical details of these procedures are described in prior publications^24,25^. Parcellations were classified according to the Desikan-Killiany Atlas^26^. The exact listing of all ROIs used can be found under supplementary material 1a. Volume was measured in mm^3^. In addition to the exploratory whole-brain approach, hypothesis-driven group comparisons were also performed with volumes of cortical regions that had been shown in a meta-analysis to be specifically affected in FMS^5^ (namely, the left medial frontal cortex and the right posterior cingulate cortex). Since the factor age has been shown to be associated with differences in white and grey matter volume^27^, we decided to include this factor as a covariate. We also included the pain intensity score of the GCPS as a covariate.

#### Structural connectivity: diffusion tensor imaging

The Oxford Centre for Functional Magnetic Resonance Imaging of the Brain software library (FSL, Oxford, UK, https://www.fmrib.ox.ac.uk)^28^ was used for DTI data analysis and preprocessing. Our diffusion data, recorded in reversed phase-encode blips, were preprocessed using the FSL tools “topup”^29^, “eddy (correction)”, “BET”^30^, and “FNIRT”. FA images and eigenvalue images were created by fitting a tensor model to the preprocessed diffusion data using the FSL FDT toolbox (Functional MRI of the Brain Diffusion Toolbox, DTIFIT). For ROI specific evaluation of the FA data we created a mask with the ICBM-DTI-81 white-matter labels atlas (Laboratory of Brain Anatomical MRI, Johns Hopkins University^31^) in the same space and calculated the average FA value of all voxels in 48 ROIs. The exact listing of all tracts used as ROIs can be found under supplementary material 1b.These data were analyzed for group comparisons with ANCOVAs including post-hoc testing (Tukey) and correlated with clinical data and questionnaires using a spearman Rho correlation for non-normally-distributed z-standardized clinical data analysis (significance level of 0.01, two-tailed, confidence interval 0.95). In addition to the exploratory whole-brain approach, hypothesis-driven group comparisons were also performed with white matter tracts that had been shown to be affected in FMS (namely the thalamus^32^, the corpus callosum^7^, the cingulum and the white matter adjacent to the insula (anterior limb of the internal capsula^33^)).

#### Functional connectivity: resting state BOLD fMRI

Resting state functional data were spatially preprocessed using SPM12 (Welcome Trust Centre for Neuroimaging, University College London, United Kingdom; http://www.fil.ion.ucl.ac.uk/spm/) and the CONN Toolbox v18 (https://www.nitrc.org/projects/conn, RRID:SCR_009550^34^) running in Matlab R2019a (The Mathworks Inc, USA). The reason for changing from FSL to the Conn Toolbox run in SPM was the extensive ROI to ROI analysis provided by this toolbox. Functional data were realigned, slice-time corrected, spatially normalized to the Montreal Neurological Institute (MNI) space, and spatially smoothed with a FWHM Gaussian kernel of 8 mm. We collected fieldmaps and undistorted the EPI images using the Fieldmap Toolbox (SPM). Motion parameters from realignment were evaluated, and a motion artefact threshold (translation > 3 mm, rotation > 1°) was employed for exclusion. Participant motion parameters were included as first-level covariates. No participants displayed gross movements to require total exclusion. Slices with motion parameters outside of the threshold were discarded. After denoising, quality control measurements (mean motion and max motion) were correlated and plotted with the functional connectivity values to control for influences (QC-FC correlations). To remove blood-oxygen-level-dependent (BOLD) signal from the cerebral white matter and ventricles, each participant’s T1-weighted MPRAGE image was automatically segmented into grey matter, white matter, cerebrospinal fluid, normalized and transformed to MNI space using the Computational Anatomy Toolbox (CAT12; http://www.neuro.uni-jena.de/cat/) running in SPM12. BOLD data were bandpass filtered (0.008–0.09 Hz) to reduce low-frequency drift and noise effects. We then generated seed-to-seed connectivity maps for each individual using 164 seeds. These seeds are provided in the CONN software^35^. The exact classification of all seeds and the MNI coordinates of all network hubs are documented in supplementary material 1c. Individual correlation maps were generated. These results were subsequently used for second-level analysis of relative functional connectivity using an ANCOVA, implemented in the CONN toolbox, to investigate differences in seed-to-seed connectivity between groups. We applied a seed-to-seed analysis to investigate which brain areas show hyper- or hypoconnectivity between patients and controls and between subgroups. In addition to the exploratory whole-brain approach, hypothesis-driven group comparisons were also performed with seed regions that had been shown to be affected by FMS (namely the insular cortex^36^, the frontoparietal network^37^, the default mode network^10^ and the somatosensory network^38^). Pain intensity (GCPS) and ADS (depression) scores were included as second-level covariates. The influence of the IENFD data on the FC-values was analyzed using a linear regression model. False discovery rate (FDR) correction was applied at the cluster level (p < 0.05).

### Statistical analysis

Data were analyzed with IBM SPSS Statistics for Windows, Version 25.0 (IBM Corp. Armonk, NY, USA) and JASP (JASP Team (2021) (Version 0.14.1, Windows 10). We tested the clinical data for normal distribution with a Shapiro–Wilk test and then, depending on the result, examined for group differences with a two-tailed t-test or a Mann–Whitney-U test. Data are given as mean ± SD or median/range unless otherwise specified. We used the Levene test with a significance threshold of 0.05 to check the data for equivalence of variance. The confidence interval was set at 95%. ROI group means of the structural, DTI and functional connectivity data were compared using an ANCOVA after controlling for interactions between the covariate and fixed factor and Tukey-tests for post-hoc comparisons. For the ANCOVA, effect sizes are displayed as ώ^2^, which is based on Cohens f^2^ (f^2^/(1 + f^2^) and Cohen’s d. The correlation analyses were performed with a Pearson correlation (after controlling for the distribution of the data), 1000 samples of bootstrapping and a significance level of 0.01. All post-hoc group comparisons were corrected for multiple comparisons using the false discovery rate algorithm^39^.

### Data availability

The raw, skull stripped, data used to analyze the following results can be obtained upon request from the corresponding author. The processing and statistical analysis of the data was done using established neuroimaging software, as described in the methods. The STROBE Statement-Checklist was used for the quality control of our case–control study.

## Results

### Patient population

The patient group (n = 43, mean age 53.5 ± 6.5 years, mean BMI 28.2 ± 5.0) and the healthy control group (n = 40, mean age 52.5 ± 6.7 years, mean BMI 26.6 ± 5.0) did not differ in age and BMI. The subgroups noPNS (normal IENFD) and PNS (decreased IENFD) differed in BMI, with a higher BMI in the PNS subgroup (Tables 1, 2).

**Table 1:** Clinical data compared between patients and controls.

	Patients all (n = 43)	Controls (n = 40)	p-value
	Mean ± SD	Mean ± SD	
Age	53.5 ± 6.5	52.6 ± 6.7	< 0.51
BMI	28.2 ± 5	26.6 ± 5	< 0.14

**Table 2:** Clinical and questionnaire data compared between subgroups.

	PNS (n = 21)	noPNS (n = 22)	p-value
	Mean ± SD/median (range)	Mean ± SD/median (range)	
Age	53.5 ± 6.7	53.4 ± 6.5	< 0.9
BMI	30.9 ± 4.2	25.5 ± 4.2	** < 0.001**
IENFD lower leg (fibers/mm)	3.9 ± 1.5	10 ± 2.6	** < 0.001**
IENFD upper thigh (fibers/mm)	5.7 ± 1.5	11.5 ± 2.8	** < 0.001**
Time since diagnosis (years)*	5 (1–19)	5 (0–14)	< 0.51
Duration of pain due to the disease (years)	16.8 ± 10.8	18. 8 ± 12.7	< 0.71
Number of tender points*	14 (11–18)	15 (7–18)	< 0.23
WPI*	13.0 (10–19)	15 (8–18)	< 0.82
SSS*	7 (6–10)	7 (5–11)	< 0.87
HbA1c (%)	5.4 ± 0.3	5.3 ± 0.2	< 0.16
Sural nerve SNAP (µV)	22.6 ± 7.8	25.1 ± 12.5	< 0.45
Sural nerve conduction velocity (m/s)	48.3 ± 4.05	50.4 ± 3.5	< 0.09
Serum vitamin D (µg/l)	30.1 ± 14.1	30.1 ± 11.1	< 0.99
Highest education level**	3 (2–5)	3 (2–5)	< 0.13
NPSI sum score	31. 1 ± 4.2	25.5 ± 4.2	< 0.09
GCPS pain intensity	73.6 ± 10.8	64 ± 15.1	** < 0.02**
GCPS disability due to pain*	66.7 (10–83.3)	53.3 (16.6–86.6)	< 0.09
Pain catastrophizing scale	26.7 ± 10.1	20.7 ± 10.3	< 0.06
ADS	27.8 ± 11.8	21.2 ± 11.4	< 0.07
FIQ	51.9 ± 12	42.3 ± 13.2	** < 0.01**
The O’Leary-Sant symptom index and problem index*	12 (0–33)	9 (1–22	< 0.28
STAI	47.1 ± 11.6	44.3 ± 13.3	< 0.48

*ADS* Allgemeine depressionskala, *BMI* body mass index, *FIQ* Fibromyalgia Impact Questionnaire, *GCPS* Graded Chronic Pain Scale, *IENFD* intraepidermal nerve fibre density, *NPSI* Neuropathic Pain Symptom Inventory, *SNAP* sensory nerve action potential, *SSS* Symptom Severity Score, *STAI* State-Trait Anxiety Inventory, *WPI* Widespread Pain Index.

*These data are not normally distributed, therefore the median and the range are shown here and a Mann–Whitney U test was applied.

** (1: Elementary school, 2: Primary school, 3: Secondary school, 4: High school, 5: University).

### Clinical data and questionnaires

We included patients with normal skin innervation and patients with reduced IENFD both at the lower leg and the upper thigh from the cohort described in^4^. In patients with reduced distal and proximal IENFD (PNS group), FMS symptoms were more severe (p = 0.02) and quality of life was lower compared to FMS patients with normal distal and proximal IENFD (p = 0.01) as reflected by the values of the GCPS pain intensity and the FIQ questionnaire (Table 2). There was no difference between the subgroups regarding parameters evaluating how widespread the pain was (WPI or tender points).

#### Structural analysis

**Figure 1:**
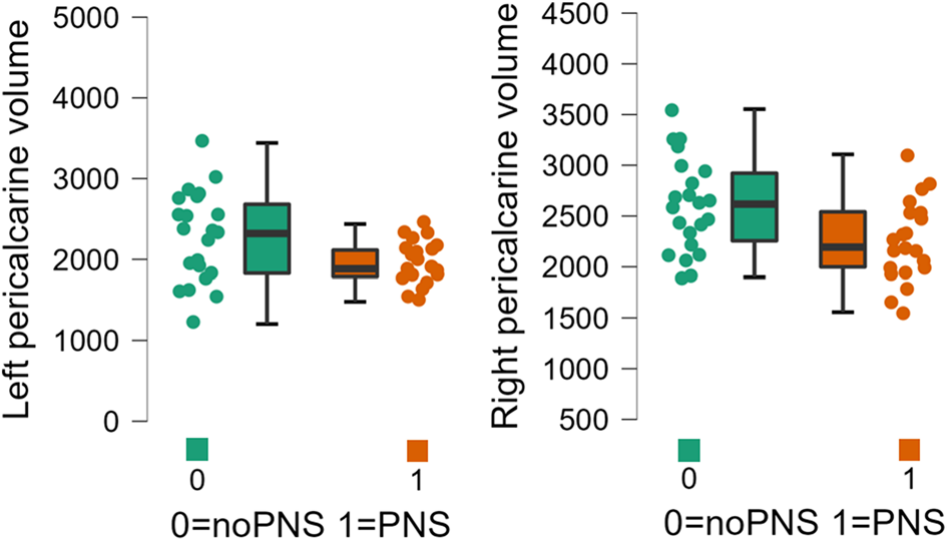
Group differences of cortical thickness. Plots showing a lower volume of the bilateral pericalcarine cortex in the PNS subgroup compared to the noPNS subgroup. Volume is measured in mm^3^.

With the values of the cortical volume per parcellation calculated by Freesurfer, we performed an ANCOVA with post-hoc testing including all patients (n = 43), PNS patients (n = 21) and noPNS patients (n = 22). Cortical volume differed between the FMS and control groups in 10 cortical regions (see Table 3). Cortical volume differed between the subgroups (PNS versus noPNS) in the left pericalcarine cortex (F = 4.1, p-adjusted = 0.049, ώ2 = 0.06) and the right pericalcarine cortex (F = 7.2, p-adjusted = 0.03, ώ2 = 0.13) (see Fig. 1). Except for the left pericalcarine cortex, all cortical regions of FMS patients showed lower volumes than those of healthy controls (see supplementary material 2).

**Table 3:** Results of cortical volume analysis after FDR-correction.

	Cortex parcellation	p-adjusted	F-value	ώ-square
Patients vs controls	Left fusiform	0.04	4,2	0,09
	Left inferiorparietal	0.04	3,6	0,08
	Left inferiortemporal	0.04	4,6	0,1
	Left insula	0.04	3,4	0,08
	Left pericalcerine	0.03	3,8	0,09
	Right middletemporal	0.01	5,4	0,12
	Right parsopercularis	0.04	3,2	0,07
	Right superiorfrontal	0.03	3,5	0,08
	Right superiortemporal	0.04	3,5	0,08
	Right supramarginal	0.04	4,6	0,1
PNS vs NoPNS	Left pericalcarine	0.049	4.1	0.06
	Right pericalcarine	0.03	7.2	0.13

To examine possible influences of clinical data including the severity of pain and depression on cortex volume, correlation analyses between questionnaire data for pain and depression and cortical volumes were calculated. This was a-priori restricted to the 10 ROIs, which showed significant alterations in the FMS group compared to the control group. We found no significant influence of clinical data including the severity of pain and depression on cortex volume in the correlation analysis after FDR correction.

#### Structural analysis of FMS “specific” regions

Following a meta-analysis that analyzed FMS data from voxel-based morphometry^5^, we explicitly tested group differences in the volume of the left medial frontal cortex, as well as the right posterior cingulate cortex. Indeed, the FMS group showed a smaller cortex volume in the left frontal pole (p = 0.03, η^2^ = 0.05), in the posterior cingulate cortex (p = 0.04, η^2^ = 0.05), and trendwise in the left rostral midfrontal cortex (p = 0.08, η^2^ = 0.04) compared to the control group. The subgroup comparison PNS versus noPNS showed no differences in these regions (left frontal pole (p = 0.26, η^2^ = 0.03), posterior cingulate cortex (p = 0.27, η^2^ = 0.03), left rostral midfrontal cortex (p = 0.6, η^2^ = 0.006)).

#### Diffusion tensor imaging

**Figure 2:**
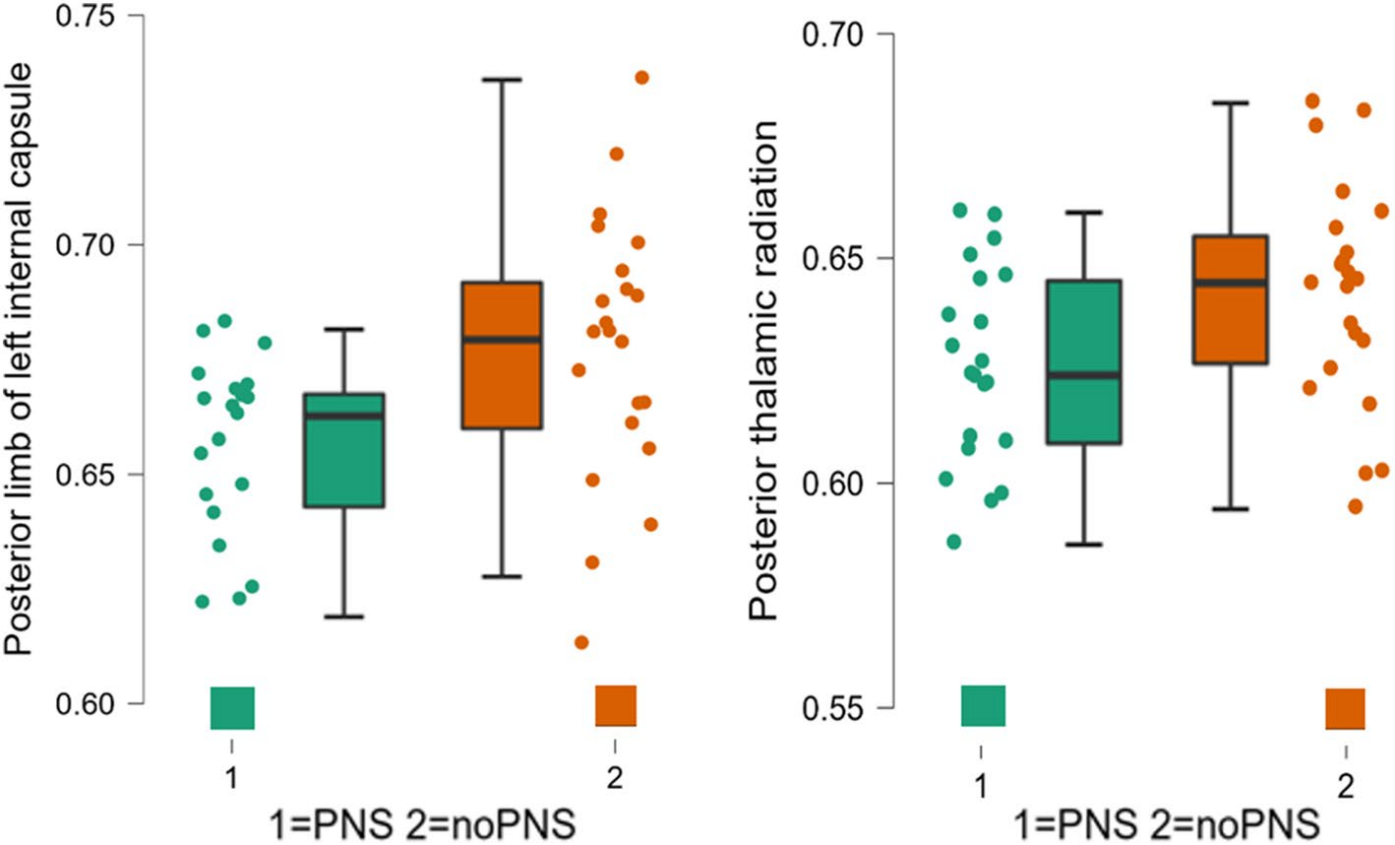
Group differences of white matter integrity. Plots showing decreased FA of two white matter tracts in the PNS subgroup compared to the noPNS subgroup.

In the ROI-based analysis comparing patients and controls, a significant increase in FA was found in 14 out of 48 ROIs in FMS patients (after FDR-correction). This was evident in corticospinal pathways such as the corona radiata, but also in regions of the limbic systems such as the fornix and cingulum. A detailed list of these regions with the respective FA values can be found in Table 4. Scatter plots to check for the distribution of the data can be found in the supplementary material 3a/b. The ROI-based comparison of the two subgroups PNS and noPNS showed elevated FA levels in the left posterior limb of the internal capsule and the posterior thalamic radiation (after FDR-correction) (Fig. 2).

**Table 4:** Between group comparisons of the FA data (ROI-wise).

	White matter tract	t-value	p-adjusted	Cohen's d	Group	N	Mean	SD	SE
Patients vs controls	Anterior corona radiata l	−3.292	0.009	−0.719	Controls	41	0.540	0.004	0.004
					Patients	43	0.556	0.003	0.003
	Body of corpus callosum	−3.705	0.004	−0.809	Controls	41	0.825	0.003	0.003
					Patients	43	0.840	0.002	0.002
	Cingulum 40	−4.384	0.001	−0.957	Controls	41	0.609	0.006	0.006
					Patients	43	0.642	0.005	0.005
	Cingulum 41	−3.456	0.009	−0.754	Controls	41	0.599	0.006	0.006
					Patients	43	0.626	0.005	0.005
	Fornix 44	−3.843	0.004	−0.839	Controls	41	0.573	0.004	0.004
					Patients	43	0.595	0.004	0.004
	Genu of corpus callosum	−2.939	0.01	−0.642	Controls	41	0.740	0.004	0.004
					Patients	43	0.757	0.004	0.004
	Pontine crossing tract	−2.992	0.02	−0.653	Controls	41	0.768	0.004	0.004
					Patients	43	0.785	0.004	0.004
	Posterior corona radiata r	−2.992	0.01	−0.642	Controls	41	0.534	0.02	0.004
					Patients	43	0.551	0.02	0.004
	Posterior limb of internal capsule l	−3.215	0.01	−0.702	Controls	41	0.650	0.004	0.004
					Patients	43	0.668	0.004	0.004
	Posterior thalamic radiation 34	−3.242	0.01	−0.708	Controls	41	0.617	0.004	0.004
					Patients	43	0.634	0.004	0.004
	Superior corona radiata l	−2.832	0.02	−0.618	Controls	41	0.551	0.005	0.005
					Patients	43	0.568	0.004	0.004
	Superior corona radiata r	−3.129	0.01	−0.683	Controls	41	0.540	0.004	0.004
					Patients	43	0.556	0.003	0.003
	Superior longitudinal fasciculus r	−2.773	0.02	−0.605	Controls	41	0.566	0.005	0.005
					Patients	43	0.584	0.004	0.004
	Uncinate fasciculus l	−0.367	0.04	−0.080	Controls	41	0.586	0.008	0.008
					Patients	43	0.590	0.007	0.007

	White matter tract	F-value	p-adjusted	ώ-square	Group	N	Mean	SD	SE
PNS vs noPNS	Posterior limb of internal capsule l	4.8	0.034	0.08	PNS	21	0.658	0.020	0.004
					NoPNS	22	0.678	0.029	0.006
	Posterior thalamic radiation 34	4.9	0.048	0.09	PNS	21	0.626	0.022	0.005
					NoPNS	22	0.642	0.025	0.005

The Pearson correlation analysis, a-priori restricted to the 14 regions that revealed differences in the group comparison, showed a negative association with the anxiety questionnaire (STAI-S) and the FA of the fornix (Pearson’s r = −0.4, p = 0.006), the posterior thalamic radiation (Pearson’s r = −0.4, p = 0.006) and the right posterior corona radiata (Pearson’s r = −0.4, p = 0.005). This means that higher anxiety scores were associated with lowered FA in the respective areas.

#### Diffusion tensor imaging of FMS “specific” regions

White matter tracts that had already shown changes in patients with FMS in the literature are the corpus callosum, the thalamus, the cingulate, and the insular cortex connecting tracts (anterior limbs of the internal capsule). Our data indicated also an increased FA in the FMS group compared to controls in the cingulum (p < 0.001, η^2^ = 0.13), in the body of the corpus callosum (p < 0.001, η^2^ = 0.14), in the genu of the corpus callosum (p = 0.004, η^2^ = 0.1), and in the posterior thalamic radiation (p = 0.002, η^2^ = 0.12). No significant differences were found in the anterior limb of the left (p = 0.2, η^2^ = 0.02) and right (p = 0.62, η^2^ = 0.003) internal capsule and the splenium of the corpus callosum (p = 0.53, η^2^ = 0.005).

Subgroup comparison between PNS and noPNS showed an increased FA of the posterior thalamic radiation in the noPNS subgroup (p = 0.03, η^2^ = 0.1). No subgroup differences were seen in the cingulum (p = 0.9, η^2^ < 0.001), body of the corpus callosum (p = 0.57, η^2^ < 0.001), genu of the corpus callosum (p = 0.99, η^2^ < 0. 001), splenium of the corpus callosum (p = 0.47, η^2^ = 0.01), and the anterior limb of the left (p = 0.7, η^2^ = 0.003) and right (p = 0.92, η^2^ < 0.001) internal capsule.

#### Functional resting state imaging

**Figure 3:**
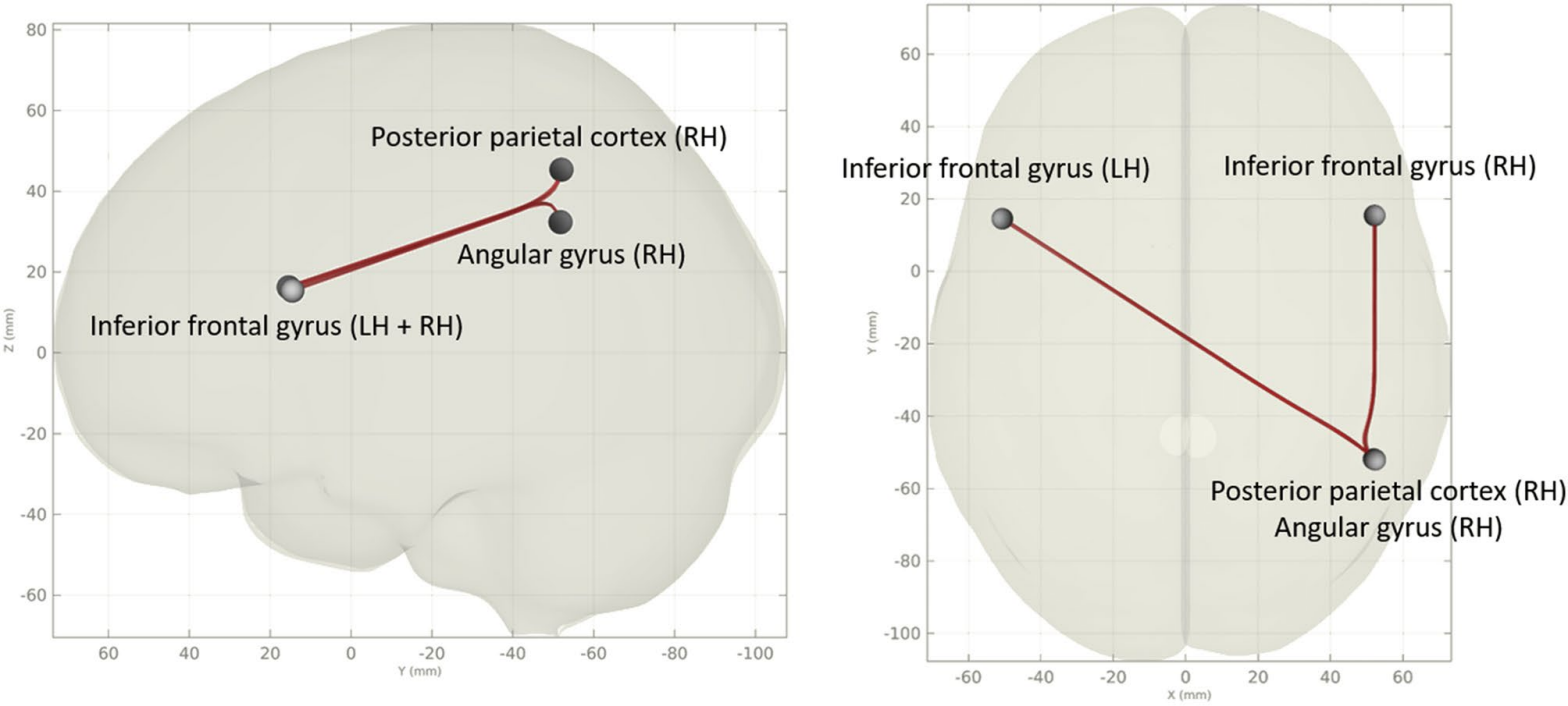
Group differences of functional seed-to-seed connectivity. Hyperconnectivity cluster in the PNS subgroup compared to the noPNS subgroup (LH: left hemisphere, RH: right hemisphere).

Seed-to-seed analysis between patients and controls showed significant hypoconnectivity of the right midfrontal gyrus to the posterior cerebellum (p-FDR = 0.048) and to the right crus cerebelli 1 (p-FDR = 0.048) in FMS patients. Seed-to-seed analysis between the subgroups noPNS and PNS showed one FDR-corrected cluster of the PNS subgroup compared to the noPNS subgroup (F = 12.8, p-adjusted = 0.049) with hyperconnectivity between the left and right inferior frontal gyrus (IFG) and the right angular gyrus (left IFG: T = 3.33; right IFG: T = 3.27) and posterior parietal cortex (left IFG: T = 2.93; right IFG: T = 3.27) respectively (Fig. 3).

The linear regression model with the IENFD values as independent variables showed no significant associations with the ROI-ROI functional connectivity after FDR-correction.

#### Functional resting state imaging of FMS “specific” network hubs

Network hubs that had already shown changes in FMS patients in previous publications are the default mode network, the somatosensory network, the frontoparietal network, and the insular cortex. Even after restricting the analysis to these regions of interest, we could not find any connectivity cluster differences between the FMS and the control group or between the PNS and noPNS subgroups in our data.

## Discussion

In this study, two group comparisons were conducted using structural, DWI and functional MRI data: Firstly, we compared FMS patients to healthy controls, secondly, we divided the FMS group into two subgroups with and without PNS pathology (PNS and noPNS groups) and compared these subgroups with each other. While the structural and functional differences in MRI studies of FM patients have been described in the literature, so far no study has investigated the possible interaction between the peripheral nervous system and the brain of FMS patients.

We show that in FMS (1) cortical volume is decreased in the left and right frontal/temporal cortices and the left insula, (2) FA is generally increased in corticospinal tracts and regions of the limbic system and (3) functional connectivity is reduced between the right midfrontal gyrus and the posterior cerebellum as well as the right crus cerebelli.

Comparison of the noPNS and PNS subgroups showed (1) lower volumes in the bilateral pericalcarine cortex in the PNS group, (2) lower FA in the left posterior limb of internal capsule and in the posterior thalamic radiation in the PNS group and (3) a hyperconnectivity cluster between the bilateral inferior frontal gyri, the angular gyrus and the posterior parietal cortex in the PNS group. In summary, the noPNS group showed greater deviations from healthy controls in structural MRI measures than the PNS group.

### Comparison of the present findings with published data

Our results on the cortical volume are for the most part (regarding the alterations in the temporal, parietal and insular cortices) in line with the results of a meta-analysis which pooled structural and functional MRI studies comparing FMS patients to healthy controls^40^. These regions also appear to change their cortical thickness as the disease progresses^41^. Decreased gray matter in the left fusiform and prefrontal cortex was also found in FMS patients in another voxel morphometry-based meta-analysis^42^. In our hypothesis-driven analysis restricted to regions that showed lower cortex volumes in a meta-analysis of structural FMS data (left medial frontal cortex and right posterior cingulate cortex), we were able to reproduce the results of the meta-analysis^5^. However, in our subgroup comparisons, these regions showed no significant differences. The prefrontal cortex is a known site of pain modulation. Indeed, a dual role has been described including antinociceptive effects by modulating sensory afferent influx, as well as the furthering of chronic pain via corticostriatal projections. Interestingly, decline of prefrontal cortex volume in chronic pain can be reversed with successful biopsychosocial therapy, be it cognitive behavioral therapy, exercise or transcranial magnetic stimulation^43^.

Our subgroup comparison of cortex volume data showed a bilateral decrease in the volume of the pericalcarine cortex in the PNS group. Interestingly, in our results, the pericalcarine cortex is the only region that shows larger volumes in the FMS patients compared to the healthy controls. Thus, the noPNS group has a greater change in pericalcarine volume compared to the healthy controls. The pericalcarine cortex is part of the visual cortex. In our literature research, this region has not yet been associated with FMS symptoms. A magnetoencephalography study showed that the visual cortex in FMS patients has decreased connectivity to other brain regions^44^. This hypoconnectivity was also demonstrated in another study using resting state fMRI^45^ and was associated with decreased resiliency towards pain^46^. However, the pericalcarine cortex is also involved in other pain disorders, for example, its volume changes during acute migraine attacks and normalizes in post-ictal phases^47^. Our results do not allow us to determine whether the pericalcarine cortices decrease in volume during the course of the disease in the PNS group or whether the difference exists at the onset of the disease. Longitudinal studies are needed to explore the role of the pericalcarine cortex in pain development.

Regarding FA, a marker for the integrity of the white matter, our whole brain analysis showed an increase in FA in the corona radiata and regions of the limbic system (e.g. fornix and cingulate cortex) in the FMS group compared to controls. The previous results of diffusion imaging in FMS patients are not consistent, and the results here vary widely. Regions that frequently showed changes in FA in the literature were the corpus callosum, the cingulum, the thalamus, and the anterior limb of the internal capsule adjacent to the insular cortex^7,33^. Except for the anterior limbs of the internal capsule, we were able to reproduce these results in our hypotheses driven analyses. Regarding our subgroups analyses, two regions showed a significant decrease of FA in the PNS group compared with the noPNS group (left posterior limb of internal capsule and the posterior thalamic radiation). Increased FA of these regions has already been found in studies with FMS patients or other chronic pain disorders and was associated with pain severity^48^. It has also been shown in FMS patients that white matter pathways with increased FA after a prolonged period of increased activity^49^, in this case in pain processing regions, show decreased FA again after pain chronification and show lower values than healthy controls^33^. Longitudinal study designs are needed to clarify the extent to which FA changes over the course of chronic pain disorders and the influences of a reduction or increase in FA on symptoms.

Regarding functional connectivity, even after limiting the regions of interest included in the analysis to network hubs already published in the FMS literature (default mode network, somatosensory network, frontoparietal network, insular cortex)^10,36,38,50^ we could not reproduce alterations in these hubs with our data. The reason for this could be the lack of control for depression or pain intensity in other studies or different methods of analysis. The cluster found in our subgroup analysis has not been described in the FMS literature before. All involved regions (inferior frontal gyrus, angular gyrus and posterior parietal cortex) are involved in attention and evaluation of external and internal stimuli. Overactivation of the angular gyrus in fMRI has been associated with a stronger negative evaluation of pain^51^, while the inferior frontal gyrus seems to be involved in the regulation of emotions^52^. The posterior parietal gyrus with its connections to the somatosensory cortex appears to have an important role in the spatial perception of pain stimuli^53^.

### Are the findings specific for FMS?

Most of our findings have been described in other publications about chronic pain imaging^54^. For example, it has already been suggested that a lower activity of the prefrontal cortex, a well-known pain modulation area, could lead to a failure in the elimination of subcortically driven fear behaviors, thereby resulting in pain chronification^55^. It is currently unclear whether these processes are adaptive, maladaptive or cause some of the symptoms. In order to better understand the pathophysiology of FMS, it is therefore important to first understand the role of brain neuroplasticity in chronic pain, as a brain signature of pain appears to be found across various pain syndromes^56^. Neuroimaging studies with multiple pain syndromes as comparison groups are needed here before finding brain regions specific to FMS that could potentially trigger some of the symptomatology.

### Limitations of our study

Our study has some limitations. Because our study was designed as a cross-sectional study, the question of the reasons for and the effects of our detected group differences cannot be answered. By including individual pain intensity as a covariate in our group statistics, we attempted to account for a possible influence of pain intensity on our MRI results. However, because none of the MRI modalities showed a significant association with IENFD scores after FDR correction, we cannot rule out the possibility that subgroup differences were driven by other factors not captured in our clinical examinations. Furthermore, even structural MRI markers, such as cortical volume, are subject to temporal variations, depending, for example, on acute stimulus severity^57^. This emphasizes the need for longitudinal studies.

The healthy controls in our study did not receive a skin biopsy, so we cannot rule out that some persons with reduced IENFD might have been in this group. However, in our previous study^4^, only 2% of normal controls had reduced IENFD at the lower and upper leg, so that it is highly unlikely that a large number of our present controls would have had this finding.

## Conclusions

While structural and functional MRI changes in FMS patients have already been investigated, our study first demonstrated differences between FMS subgroups with and without peripheral nerve involvement. The study design obviously does not allow any conclusions to be drawn about the reasons for and effects of these subgroup differences. While most clinical trials on FMS therapy included only patients diagnosed according to current diagnostic criteria, one has to consider that FMS is a heterogeneous condition with potentially different underlying pathophysiological processes within subgroups. These subgroups might respond differentially to specific treatments. Psychiatric comorbidities, such as depression and anxiety, also affect the brain structure in FMS and thus influence the results in MRI imaging. We therefore advocate that future studies should take into account the different subgroups of patients both on the basis of small nerve fiber pathology, symptom severity, and psychiatric comorbidities.

## Supplementary Information

Supplementary Information.
