# Supplementary material for: CNS imaging characteristics in fibromyalgia patients with and without peripheral nerve involvement
Source: Sci Rep. 2022 Apr 25;12:6707. doi: 10.1038/s41598-022-10489-1 (PMC9038916; doi:10.1038/s41598-022-10489-1)
Supplement: Supplementary file 1 — Supplementary Information. [file 41598_2022_10489_MOESM1_ESM.docx]

**Supplementary material**

**Supplementary material 1a:**

***Lobes included by the Desikan-Killiany atlas ROIs***

**Frontal**

- Superior Frontal
- Rostral and Caudal Middle Frontal
- Pars Opercularis, Pars Triangularis, and Pars Orbitalis
- Lateral and Medial Orbitofrontal
- Precentral
- Paracentral
- Frontal Pole

**Parietal**

- Superior Parietal
- Inferior Parietal
- Supramarginal
- Postcentral
- Precuneus

**Temporal**

- Superior, Middle, and Inferior Temporal
- Banks of the Superior Temporal Sulcus
- Fusiform
- Transverse Temporal
- Entorhinal
- Temporal Pole
- Parahippocampal

**Occipital**

- Lateral Occipital
- Lingual
- Cuneus
- Pericalcarine

**Cingulate**

- Rostral Anterior (Frontal)
- Caudal Anterior (Frontal)
- Posterior (Parietal)
- Isthmus (Parietal)

**Supplementary material 1b:**

***Tracts included in the ICBM DTI-81 Atlas:***

**Tracts in the brainstem:**

- Corticospinal tracts (CST)
- Medial lemniscus (ML)
- Medial longitudinal fasciculus (MLF)
- Inferior cerebellar peduncle (ICP)
- Middle cerebellar peduncle (MCP)
- Superior cerebellar peduncle (SCP)

**Projection fibers:**

- Corona radiata
- Anterior limb of internal capsule (ALIC)
- Posterior limb of internal capsule (PLIC)
- Retrolenticular part of the internal capsule (RLIC)
- Cerebral peduncle (CP)
- Posterior thalamic radiation

**Association fibers:**

- Superior longitudinal fasciculus (SLF)
- Superior fronto-occipital fasciculus (SFO)
- Uncinate fasciculus (UNC)
- Inferior fronto-occipital fasciculus (IFO) / Uncinate fasciculus (UNC)
- Inferior fronto-occipital fasciculus (IFO) / Inferior longitudinal fasciculus (ILF)
- Sagittal Stratum (SS)
- External capsule (EC)
- Cingulum (CG)
- Fornix (FX) and Stria terminalis (ST)

**Commissural fibers:**

- Anterior commissure (AC)
- Corpus callosum (CC)
- Tapetum (TAP)

**Supplementary material 1c:**

***Regions of interest in the uninformed functional connectivity analyses:***

**Atlas regions:**

FP r (Frontal Pole Right)

FP l (Frontal Pole Left)

IC r (Insular Cortex Right)

IC l (Insular Cortex Left)

SFG r (Superior Frontal Gyrus Right)

SFG l (Superior Frontal Gyrus Left)

MidFG r (Middle Frontal Gyrus Right)

MidFG l (Middle Frontal Gyrus Left)

IFG tri r (Inferior Frontal Gyrus, pars triangularis Right)

IFG tri l (Inferior Frontal Gyrus, pars triangularis Left)

IFG oper r (Inferior Frontal Gyrus, pars opercularis Right)

IFG oper l (Inferior Frontal Gyrus, pars opercularis Left)

PreCG r (Precentral Gyrus Right)

PreCG l (Precentral Gyrus Left)

TP r (Temporal Pole Right)

TP l (Temporal Pole Left)

aSTG r (Superior Temporal Gyrus, anterior division Right)

aSTG l (Superior Temporal Gyrus, anterior division Left)

pSTG r (Superior Temporal Gyrus, posterior division Right)

pSTG l (Superior Temporal Gyrus, posterior division Left)

aMTG r (Middle Temporal Gyrus, anterior division Right)

aMTG l (Middle Temporal Gyrus, anterior division Left)

pMTG r (Middle Temporal Gyrus, posterior division Right)

pMTG l (Middle Temporal Gyrus, posterior division Left)

toMTG r (Middle Temporal Gyrus, temporooccipital part Right)

toMTG l (Middle Temporal Gyrus, temporooccipital part Left)

aITG r (Inferior Temporal Gyrus, anterior division Right)

aITG l (Inferior Temporal Gyrus, anterior division Left)

pITG r (Inferior Temporal Gyrus, posterior division Right)

pITG l (Inferior Temporal Gyrus, posterior division Left)

toITG r (Inferior Temporal Gyrus, temporooccipital part Right)

toITG l (Inferior Temporal Gyrus, temporooccipital part Left)

PostCG r (Postcentral Gyrus Right)

PostCG l (Postcentral Gyrus Left)

SPL r (Superior Parietal Lobule Right)

SPL l (Superior Parietal Lobule Left)

aSMG r (Supramarginal Gyrus, anterior division Right)

aSMG l (Supramarginal Gyrus, anterior division Left)

pSMG r (Supramarginal Gyrus, posterior division Right)

pSMG l (Supramarginal Gyrus, posterior division Left)

AG r (Angular Gyrus Right)

AG l (Angular Gyrus Left)

sLOC r (Lateral Occipital Cortex, superior division Right)

sLOC l (Lateral Occipital Cortex, superior division Left)

iLOC r (Lateral Occipital Cortex, inferior division Right)

iLOC l (Lateral Occipital Cortex, inferior division Left)

ICC r (Intracalcarine Cortex Right)

ICC l (Intracalcarine Cortex Left)

MedFC (Frontal Medial Cortex)

SMA r (Juxtapositional Lobule Cortex -formerly Supplementary Motor Cortex- Right)

SMA L(Juxtapositional Lobule Cortex -formerly Supplementary Motor Cortex- Left)

SubCalC (Subcallosal Cortex)

PaCiG r (Paracingulate Gyrus Right)

PaCiG l (Paracingulate Gyrus Left)

AC (Cingulate Gyrus, anterior division)

PC (Cingulate Gyrus, posterior division)

Precuneous (Precuneous Cortex)

Cuneal r (Cuneal Cortex Right)

Cuneal l (Cuneal Cortex Left)

FOrb r (Frontal Orbital Cortex Right)

FOrb l (Frontal Orbital Cortex Left)

aPaHC r (Parahippocampal Gyrus, anterior division Right)

aPaHC l (Parahippocampal Gyrus, anterior division Left)

pPaHC r (Parahippocampal Gyrus, posterior division Right)

pPaHC l (Parahippocampal Gyrus, posterior division Left)

LG r (Lingual Gyrus Right)

LG l (Lingual Gyrus Left)

aTFusC r (Temporal Fusiform Cortex, anterior division Right)

aTFusC l (Temporal Fusiform Cortex, anterior division Left)

pTFusC r (Temporal Fusiform Cortex, posterior division Right)

pTFusC l (Temporal Fusiform Cortex, posterior division Left)

TOFusC r (Temporal Occipital Fusiform Cortex Right)

TOFusC l (Temporal Occipital Fusiform Cortex Left)

OFusG r (Occipital Fusiform Gyrus Right)

OFusG l (Occipital Fusiform Gyrus Left)

FO r (Frontal Operculum Cortex Right)

FO l (Frontal Operculum Cortex Left)

CO r (Central Opercular Cortex Right)

CO l (Central Opercular Cortex Left)

PO r (Parietal Operculum Cortex Right)

PO l (Parietal Operculum Cortex Left)

PP r (Planum Polare Right)

PP l (Planum Polare Left)

HG r (Heschl's Gyrus Right)

HG l (Heschl's Gyrus Left)

PT r (Planum Temporale Right)

PT l (Planum Temporale Left)

SCC r (Supracalcarine Cortex Right)

SCC l (Supracalcarine Cortex Left)

OP r (Occipital Pole Right)

OP l (Occipital Pole Left)

Thalamus r

Thalamus l

Caudate r

Caudate l

Putamen r

Putamen l

Pallidum r

Pallidum l

Hippocampus r

Hippocampus l

Amygdala r

Amygdala l

Accumbens r

Accumbens l

Brain-Stem

Cereb1 l (Cerebelum Crus1 Left)

Cereb1 r (Cerebelum Crus1 Right)

Cereb2 l (Cerebelum Crus2 Left)

Cereb2 r (Cerebelum Crus2 Right)

Cereb3 l (Cerebelum 3 Left)

Cereb3 r (Cerebelum 3 Right)

Cereb45 l (Cerebelum 4 5 Left)

Cereb45 r (Cerebelum 4 5 Right)

Cereb6 l (Cerebelum 6 Left)

Cereb6 r (Cerebelum 6 Right)

Cereb7 l (Cerebelum 7b Left)

Cereb7 r (Cerebelum 7b Right)

Cereb8 l (Cerebelum 8 Left)

Cereb8 r (Cerebelum 8 Right)

Cereb9 l (Cerebelum 9 Left)

Cereb9 r (Cerebelum 9 Right)

Cereb10 l (Cerebelum 10 Left)

Cereb10 r (Cerebelum 10 Right)

Ver12 (Vermis 1 2)

Ver3 (Vermis 3)

Ver45 (Vermis 4 5)

Ver6 (Vermis 6)

Ver7 (Vermis 7)

Ver8 (Vermis 8)

Ver9 (Vermis 9)

Ver10 (Vermis 10)

**Network hubs:**

DefaultMode.MPFC (1,55,-3)

DefaultMode.LP (L) (-39,-77,33)

DefaultMode.LP (R) (47,-67,29)

DefaultMode.PCC (1,-61,38)

SensoriMotor.Lateral (L) (-55,-12,29)

SensoriMotor.Lateral (R) (56,-10,29)

SensoriMotor.Superior (0,-31,67)

Visual.Medial (2,-79,12)

Visual.Occipital (0,-93,-4)

Visual.Lateral (L) (-37,-79,10)

Visual.Lateral (R) (38,-72,13)

Salience.ACC (0,22,35)

Salience.AInsula (L) (-44,13,1)

Salience.AInsula (R) (47,14,0)

Salience.RPFC (L) (-32,45,27)

Salience.RPFC (R) (32,46,27)

Salience.SMG (L) (-60,-39,31)

Salience.SMG (R) (62,-35,32)

DorsalAttention.FEF (L) (-27,-9,64)

DorsalAttention.FEF (R) (30,-6,64)

DorsalAttention.IPS (L) (-39,-43,52)

DorsalAttention.IPS (R) (39,-42,54)

FrontoParietal.LPFC (L) (-43,33,28)

FrontoParietal.PPC (L) (-46,-58,49)

FrontoParietal.LPFC (R) (41,38,30)

FrontoParietal.PPC (R) (52,-52,45)

Language.IFG (L) (-51,26,2)

Language.IFG (R) (54,28,1)

Language.pSTG (L) (-57,-47,15)

Language.pSTG (R) (59,-42,13)

Cerebellar.Anterior (0,-63,-30)

Cerebellar.Posterior (0,-79,-32)

**
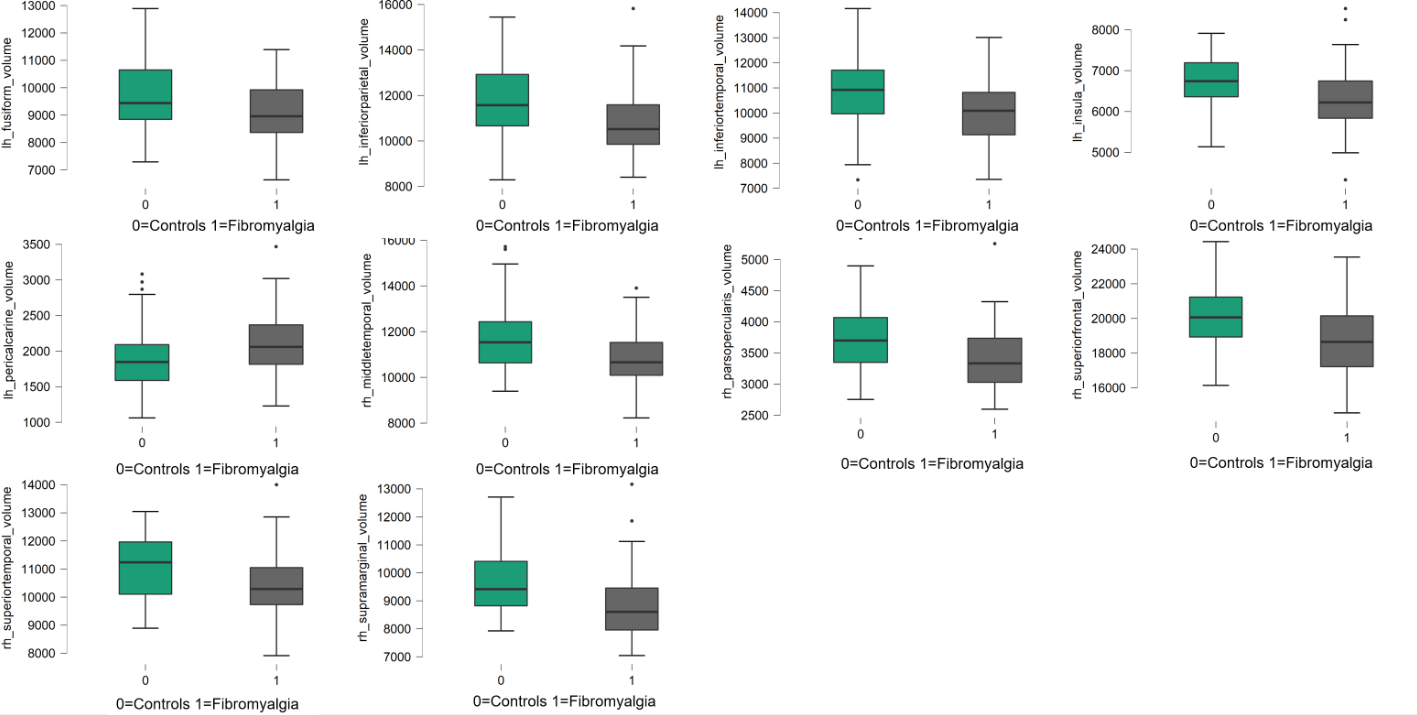
**

**Supplementary material 2:** Boxplots showing the significant alterations in cortical volumes (mm^3^) per ROI between the FMS and healthy control group.


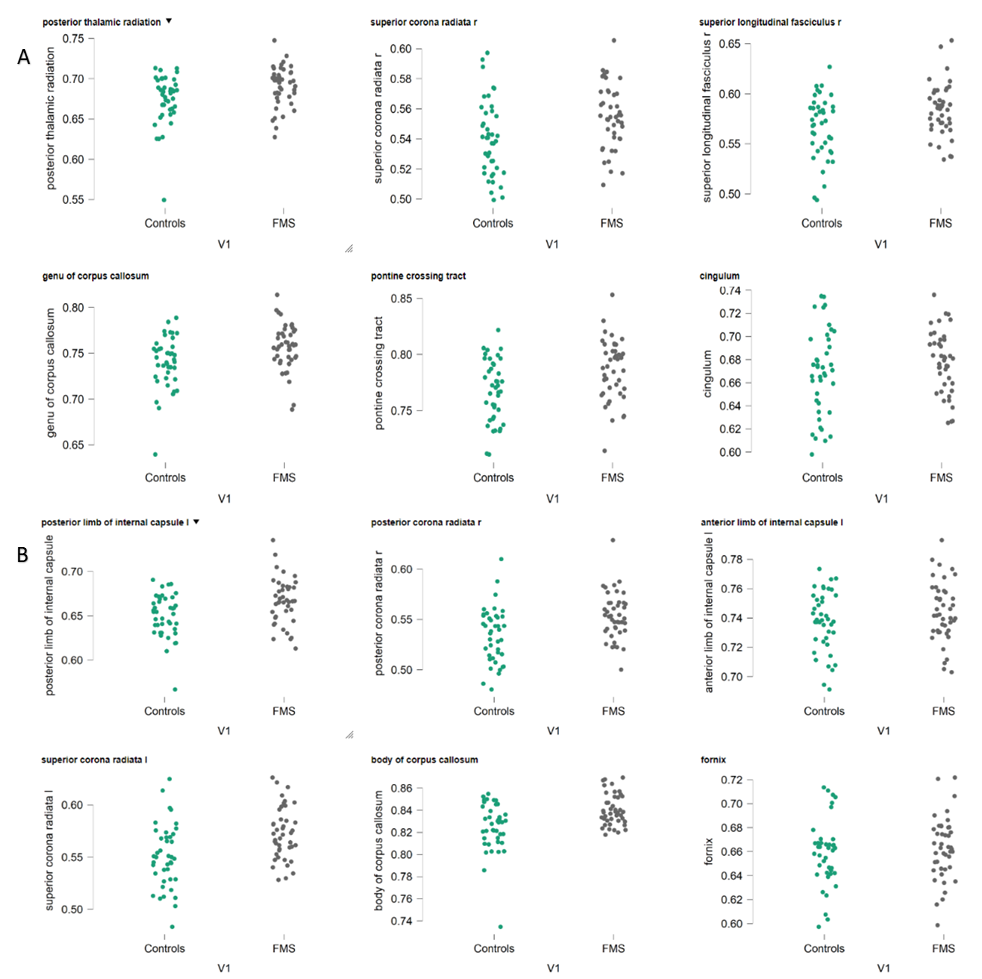


**Supplementary material 3a/b:** Scatter plots showing the significant alterations in FA per ROI between the FMS and healthy control group.
